# Balance in the elderly

**DOI:** 10.1016/S1808-8694(15)31326-4

**Published:** 2015-10-20

**Authors:** Sheelen Larissa Ruwer, Angela Garcia Rossi, Larissa Fortunato Simon

**Affiliations:** ^1^ Specialization in Speech and Hearing Therapy, Major in Hearing. Master studies in Human Communication Disorders: Hearing under course; ^2^ Ph.D. in Human Communication Disorders, UNIFESP - EPM; Joint Professor, Department of Otorhinolaryngology and Speech and Hearing Therapy, UFSM; ^3^ Speech and Hearing Therapist. Study carried out at Ambulatory of Otoneurology, Hospital Universitário de Santa Maria - HUSM

**Keywords:** elderly, balance, vestibular evaluation

## Abstract

**T**hroughout the years, the human organism goes through natural aging, having functional and structural changes. The part responsible for the corporal balance system also suffers from the aging process, creating great impact for the elderly. **Aim**: Thus, the present paper aims at studying the vestibular function of old people suffering from dizziness, tinnitus and hearing impairment. **Study design**: transversal cohort. **Material and method**: Eighty elderly individuals from two different groups were evaluated: group A - comprising 38 women and 2 men who belonged to an elderly group from Santa Maria, RS; and group B - comprising 35 women and 5 men with complaints of balance disorders. **Results**: Both groups underwent anamnesis (directed to aspects concerning dizziness, tinnitus and hearing impairment), and vestibular function evaluation (by using the computerized system of vecto-electronystagmography SCV 5.0). The results showed statistical significant difference between both groups, concerning the complaints of dizziness and tinnitus, which were more prevalent in group B. The computerized eletronystagmography revealed that most individuals had normal diagnosis; however, there was predominance of vestibular disorders in the elderly, such as Deficit Peripheral Vestibular Syndrome and Irritative Peripheral Vestibular Syndrome. **Conclusion**: It was concluded that vestibular disorders, according to vectoelectronystagmography, and complaints of dizziness, tinnitus and hearing impairment, were numerically similar in both studied groups.

## INTRODUCTION

Improvement in healthcare conditions and the increasing life expectancy in the world, as well as in Brazil, has resulted in the increase of the number of elderly, leading to increased incidence of diseases related to elderly years.

Contributions of modern medicine for the geriatric population are priceless, controlling the diseases related with this age range and favoring the increase in average life expectancy.

As years go by, human body goes through a natural process of aging, generating functional and structural modifications, reducing vitality and favoring the onset of diseases: the most prevalent are sensorial affections, bone and cardiovascular diseases and diabetes^1^.

Aging impairs the central nervous system capability to process vestibular, visual and proprioceptive signals responsible for maintaining body balance, as well as for reducing the capacity of modifying adaptative reflexes. These degenerative processes are responsible for the occurrence of vertigo and/or dizziness (presbyvertigo) and imbalance (presbytaxia) in the geriatric population.

Dizziness is an extremely frequent symptom in the world, present in all age ranges, especially in adults and the elderly. Up to the age of 65 years, dizziness is considered the second most prevalent symptom in the world. After this age, it is the most common symptom. In subjects aged over 75 years, the prevalence is as high as 80%^2^.

One of the main factors that currently limit the life of the elderly is imbalance. In 80% of the cases it can not be attributed to a specific cause, but rather to an involvement of the balance system as a whole. In over half of the cases, imbalance originates between the ages of 65 and 75 years, approximately, and about 30% of the elderly patients have the symptoms at this age. Falls are the most dangerous consequence of imbalance and difficulty to move, followed by fractures, which render the elderly bedridden for days or months, responsible for 70% of the accidental deaths in people aged over 75 years^3^.

Manifestations of body balance disorders have major impact in the elderly, which can lead to reduction of their social autonomy, given that they have to reduce their daily life activities, because of the predisposition to falls and fractures, bringing suffering, body immobility, fear to fall again and high costs to the healthcare system.

Knowing that the occurrence of dizziness (rotation or not), imbalance and falls is frequent in the elderly, it is important to assess their vestibular function so as to detect diagnostic, prognostic, prophylactic and therapeutic implications in this population.

The present study intended to study the vestibular function in two groups of elderly patients based on their complaints of dizziness, tinnitus and hearing disorders.

## MATERIAL AND METHOD

The study was developed at the Ambulatory of Otoneurology, Hospital Universitário de Santa Maria (HUSM), where we assessed 80 patients aged 60 years or over, according to Administrative Rule nº 1.395/GM, Elderly Healthcare National Policy (1999)^4^.

The 80 elderly subjects comprised two different groups characterized as follows:

Group A: comprising 40 subjects that belonged to an elderly group in the city of Santa Maria, RS - Group Mexe Coraçã o - in which participants performed weekly activities, formed by 38 female and 2 male subjects;
Group B: comprising 40 subjects who complained of body balance disorders and came to the Ambulatory of Otoneurology, HUSM by medical indication, formed by 35 female and 5 male subjects.

Inclusion criteria were:

• age 60 years or over (Administrative Rule nº 1.395/GM, Elderly Healthcare National Policy, 1999)^4^;
• to participate in the Group Mexe Coraçã o, in Santa Maria - RS, or to have been submitted to vestibular assessment at HUSM by medical indication, between October 2002 and October 2003, with previous consent to join the study.

Following current concepts of research ethics that involve human beings, we included only subjects that freely agreed to participate after they had been properly informed about the project and signed the Free Informed Consent Term.

We performed clinical ENT examination to exclude any specific ear, nose and/or throat affections.

Patients were submitted to anamnesis and vestibular assessment. In the anamnesis, we investigated aspects related to dizziness, tinnitus and hearing disorder. Vestibular assessment was carried out using a computerized vectoelectronystagmography system (VENG) SCV 5.0, brand Contronic, which can assess vestibular-oculomotor function using triangular positioning of electrodes placed close to the eyes, which record variation of corneal-retinal potential during eye movement. They are basically directed to nystagmus recording, which is the most interesting aspect for otoneurological purposes.

**Figure 1:**
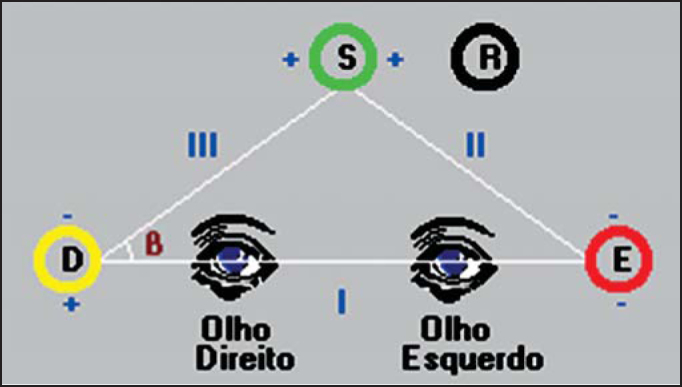

We performed previous cleaning of the periorbital skin on each side, applying electrolytic paste to the three active electrodes and to one neutral electrode, which were fixed with adhesive tape, placed as shown in Figure 1. The grounding electrode was fixed to the frontal region, the upper electrode to the midline, two centimeters above the glabella, and the other two electrodes were fixed to each canthus of the eye.

The examined patients were instructed to avoid alcoholic drinks, non-essential drugs, coffee, tea or chocolate and smoking for 24 hours before the conduction of the vestibular exam, and to fast for 3 hours before the assessment.

For statistical purposes, we used PEARSON Chi-square test, and adopted the significance level of 0.05 or 5% to compare all Groups.

## RESULTS

By analyzing the data, we could observe that concerning presence of dizziness (Table 1) and tinnitus (Table 2), there was statistically significant difference between the groups, in which we detected higher incidence of dizziness and tinnitus complaints among patients in group B.

**Table 1:** Distribution of elderly subjects in groups A and B according to presence or absence of dizziness

GROUP	A		B		TOTAL	
	N	%	N	%	N	%
With dizziness	22	55.00	38	95.00	60	75.00
Without dizziness	18	45.00	2	5.00	20	25.00
TOTAL	40	100.00	40	100.00	80	100.00

Chi-square test p = 0.0001*

* = statistically significant value

**Table 2:** Distribution of elderly subjects in groups A and B according to presence or absence of tinnitus

GROUP	A		B		TOTAL	
	N	%	N	%	N	%
With tinnitus	19	47.50	28	70.00	47	58.75
Without tinnitus	21	52.50	12	30.00	33	41.25
TOTAL	40	100.00	40	100.00	80	100.00

Chi-square test p = 0.0410*

* = statistically significant value

As to presence or absence of hearing complaint (Table 3), there was no statistically significant difference between the groups. Even though not significant, we observed that Group B presented higher values than Group A concerning this complaint.

**Table 3:** Distribution of elderly subjects in groups A and B according to presence of hearing disorder

GROUP	A		B		TOTAL	
	N	%	N	%	N	%
With hearing loss	20	50.00	23	57.50	43	53.75
Without hearing loss	20	50.00	17	42.50	37	46.25
TOTAL	40	100.00	40	100.00	80	100.00

Chi-square test p = 0.5011

We detected that the results obtained with calibration were regular in 100% (n = 80) of the subjects in both Groups.

As to spontaneous nystagmus (closed and opened eyes) and directional nystagmus, we detected absence of both in all studies subjects.

Upon studying Horizontal Pendular Tracking (RPh) (Tables 4) most of the elderly in both groups had RPh types I and II, with type I RPh more prevalent in Group B and type II in Group A. The statistical analysis did not produce significant association between type of RPh and the Groups. In the investigation of ventricular pendulum tracking (RPv) (Table 5) we could observe that in this test, type I RPv was more prevalent in elderly people in Group B, type III RPv was more prevalent in elderly in Group A, and most of the subjects in both Groups had type II RPv. Data analysis revealed statistically significant differences between the groups. In the present study, we did not consider type II pendulum tracking as a signal of central involvement because visual disorders may interfere in the analysis of this test.

**Table 4:** Distribution of elderly subjects according to groups A and B and presence of Horizontal Pendulum Tracking

GROUP	A		B		TOTAL	
	N	%	N	%	N	%
Type I	7	17.50	26	65.00	33	45.25
Type II	28	70.00	13	32.50	41	51.25
Type III	5	12.50	1	2.50	6	7.50
Type IV	0	0.00	0	0.00	0	0.00
TOTAL	40	100.00	40	100.00	80	100.00

Chi-square test p = 0.5011

**Table 5:** Distribution of elderly subjects according to groups A and B and presence of Vertical Pendulum Tracking

GROUP	A		B		TOTAL	
	N	%	N	%	N	%
Type I	1	2.50	17	42.50	18	22.50
Type II	23	57.50	22	55.00	55	56.25
Type III	16	40.00	1	2.50	17	21.25
Type IV	0	0.00	0	0.00	0	0.00
TOTAL	40	100.00	40	100.00	80	100.00

Chi-square test p = 0.0001*

* = statistically significant value

Results from Horizontal Optokinetic Nystagmus led us to realizing that most elderly patients in both groups presented symmetry and there was no statistically significant difference in the comparison between the groups.

Concerning the results obtained in PRPD (peri-rotatory nystagmus - NPR) (Table 7), we observed that in this task most of the studied subjects presented symmetry of NRP, more predominant in group A.

**Table 7:** Distribution of elderly subjects according to groups A and B and presence of Peri-Rotation Nystagmus

GROUP	A		B		TOTAL	
	N	%	N	%	N	%
Symmetrical	40	100.00	36	90.00	76	95.00
PD to the R	0	0.00	1	2.50	1	1.25
PD to the L	0	0.00	2	5.00	2	2.50
Arrefl. Bilat.	0	0.00	1	2.50	1	1.25
TOTAL	40	100.00	40	100.00	80	100.00

Not applicable x^2^

Arrefl. Bilat.= Bilateral Arreflexia

PD to the R = Directional predominance to the right

PD to the L = Directional predominance to the left

As per Post-caloric nystagmus (Table 8) most elderly patients presented normal reflex response to caloric test.

**Table 8:** Distribution of elderly subjects according to groups A and B and presence of Post-caloric Nystagmus

GROUP	A		B		TOTAL	
	N	%	N	%	N	%
Normorreflexia	30	75.00	30	75.00	60	75.00
PD to the R	3	7.50	2	5.00	5	6.25
PL to the R	1	2.50	3	7.50	4	5.00
PL to the L	4	10.00	3	7.50	7	8.75
Hyperreflexia	1	2.50	1	2.50	2	2.50
Arrefl. Bilat.	0	0.00	1	2.50	1	1.25
Did not perform	1	2.50	0	0.00	1	1.25
TOTAL	40	100.00	40	100.00	40	100.00

Chi-square test p = 0.9830

Arrefl. Bilat.= Bilateral Arreflexia

PD to the R = Directional predominance to the right

PL to the R = Labyrinthic predominance to the right

PL to the L = Labyrinthic predominance to the left

However, a reasonable number of elderly patients presented abnormalities in the test, and the most frequent affection was labyrinthic predominance, present in 13.75% (n = 16) of the elderly. There was no statistically significant association in the comparisons between the groups.

The results obtained in the vestibular assessment using computerized VENG (Table 9) demonstrated that most elderly patients presented normal diagnosis. However, there were some cases of vestibular disorders in the elderly, with predominance of deficit peripheral vestibular syndrome and irritative peripheral vestibular syndrome. We did not observe pathognomonic signals of central affections in the vestibular exam. Statistical analysis demonstrated that there were no statistically significant associations in group comparisons.

**Table 9:** Distribution of elderly subjects according to groups A and B and conclusion of vectoelectronystagmographic exam

GROUP	A		B		TOTAL	
	N	%	N	%	N	%
Normal	31	77.50	28	70.00	59	73.75
SVP D to the R, comp	4	10.00	2	5.00	6	7.50
SVP D to the L, comp	1	2.50	4	10.00	5	6.25
SVP D to the R, desc.	0	0.00	1	2.50	1	1.25
SVP I	4	10.00	5	12.50	9	11.25
TOTAL	40	100.00	40	100.00	80	100.00

Chi-square test p = 1.000

SVP D to the R, comp. = Deficit peripheral vestibular syndrome to the right, compensated

SVP D to the L, comp. = Deficit peripheral vestibular syndrome to the left, compensated

SVP D to the R, desc. = Deficit peripheral vestibular syndrome to the right, decompensate

SVP I = Irritative peripheral vestibular syndrome

## DISCUSSION

We would like to emphasize that the data in the present study were confronted only with the literature that addressed vestibular affections in the elderly in general, considering that the studied literature did not present any report on elderly subjects such as the one we had in our groups.

We detected higher incidence of dizziness complaint in subjects in group B. We may infer that the complaint of dizziness was less frequent in those that presented an active life, who had social, physical and intellectual activity. The results we found confirm the studied literature in which it was reported that vertigo is a symptom that affected 61% of all people aged over 70 years, present in 50% to 60% of the elderly people who live at home or in 81% to 91% of the elderly seen in geriatric outpatient units^5^. Other estimates point to the fact that one in each ten people in the world have or has had dizziness. Up to 65 years, it would be the most common symptom. In subjects aged over 75 years, the prevalence would be of about 80%^6^. The highest prevalence of dizziness in elderly subjects would be owed to high sensitivity of auditory and vestibular systems to clinical problems located in other parts of the body and to the process of functional deterioration of these systems resultant from aging^7^.

The tinnitus complaint had its higher incidence also in Group B subjects. Thus, similarly to the dizziness complaint, we could infer that the incidence of tinnitus complaint is higher in subjects that have little physical, social and intellectual activity. The results were similar to those found in the studied literature that showed incidence of tinnitus of about 79.4% in the geriatric population^6^. The statistics of the National Institute of Health (USA)^7^ demonstrated the prevalence of tinnitus complaints (17%) in the population of patients that came to the institution, especially the elderly. There is consensus in the literature about the relevant occurrence of complaints such as tinnitus in elderly patients^6^.

**Table 6:** Distribution of elderly subjects according to groups A and B and presence of Horizontal Optokinetic Nystagmus

GROUP	A		B		TOTAL	
	N	%	N	%	N	%
Symmetrical	40	100.00	37	92.50	77	96.25
Asymmetrical	0	0.00	3	7.50	3	3.75
TOTAL	40	100.00	40	100.00	80	100.00

Chi-square test p = 0.6080

As to presence or absence of hearing loss complaint, even though there was no statistically significant difference between the groups, we could observe that Group B presented higher values than Group A. This study is similar to the studied literature because it shows prevalence of hearing loss complaint of 13% of the population that go to institutions, especially to the elderly^7^. There is consensus in the literature that the occurrence of dizziness, imbalance and falls are the most common complaints of the elderly. Complaints such as tinnitus, difficulty to speak in noisy environment, difficulty to perceive high sounds and intolerance to loud sounds are also common and can follow such manifestations2., 6..

The results obtained with the Calibration were regular to all subjects in both groups, which is in agreement with the studied literature6., 8., 9..

For Spontaneous (opened and closed eyes) and Directional Nystagmus, we observed absence of both types in all studied elderly subjects, similar to studies carried out by other authors6., 8..

As to pendulum tracking test, we could observe that both for horizontal and vertical tasks, most of the elderly presented type II pendular nystagmus. We also detected high incidence of type III pendular nystagmus, especially in Group A, a fact that is not explained by the studied literature, opening the issue for further studies. The highest incidence of pendular nystagmus types II and III is in agreement with the literature in which pendular nystagmus in the elderly is normally of types II and III^10^. Another study that confirms these findings demonstrated that they had type III pendulum tracking in 17.6% of the elderly that comprised their study^6^. Based on such findings, we could infer that aging of the body impairs body muscle force, in special the extrinsic eye muscles, which may impair ocular tracking, presented with notches^10^. Such findings in pendulum tracking in the elderly also show that aging should be considered in the analysis of oculomotor tests^6^.

Horizontal optokinetic nystagmus was symmetrical in most of the elderly subjects, in both groups, knowing that Optokinetic nystagmus in general is symmetrical and present gains in the normal range or is slightly decreased in peripheral vestibular pathologies^11^. The findings by other authors are similar to ours, in which they found symmetry of optokinetic nystagmus in all assessed patients^6^. The literature reports presence of asymmetrical optokinetic nystagmus in 22.8% of the patients and not coordinated in 8.5% of the cases^10^.

As to the results obtained in PRPD - Peri-rotation nystagmus - we observed that only 5% of the assessed elderly had abnormalities to this type of nystagmus, which was similar to other studies that showed abnormalities in only 2.9% of the patients, characterized by the directional predominance of peri-rotation nystagmus^6^.

The Caloric test - post-caloric nystagmus - produced most normal reflex results among the elderly; however, there was a considerable number of cases that had abnormal result in the test, and the most frequent finding was labyrinthic predominance in 13.75% (n = 16). This result is similar to that of other studies, which stated that unilateral hyporreflexia of post-caloric nystagmus was a common affection to vestibular assessment of elderly patients6., 9., 12..

The results obtained from the vestibular assessment carried out with computerized VENG demonstrated that most elderly subjects presented normal diagnosis. However, there were some cases of vestibular affections in the elderly, with prevalence of deficit peripheral vestibular syndrome and irritative peripheral vestibular syndrome. We did not observe any pathognomonic signals of central affection in the vestibular exam. This finding is similar to those by other authors, which did not find central involvement in the elderly either6., 8., 13., 14.. Some authors found 9% of elderly patients with dysrythmia, which would be resultant from cerebellum affection and quadratic waves in 14%, suggesting diffuse brainstem lesion^10^.

As to type of vestibular affection, some authors described high incidence of irritative abnormalities^13^, or similar proportion of irritative and deficit peripheral vestibular syndrome^9^, or still marked predominant frequency of deficit type^6^. In this study, concerning affections found by VENG, there were similar proportions of deficit and irritative peripheral syndromes.

## CONCLUSION

Based on the critical analysis of the results, we concluded that:

• Complaints of tinnitus, hearing loss and in special dizziness largely affect elderly subjects, especially the ones that do not have an active life, as opposed to those that perform different activities in elderly groups.
• Vestibular affections detected by vectoelectronystagmography resulting from complaints of dizziness, tinnitus and auditory difficulty are numerically similar in the elderly group and in the group of elderly subjects with effective complaints of body imbalance.
